# Spray-Dried Amorphous Solid Dispersions of Griseofulvin in HPC/Soluplus/SDS: Elucidating the Multifaceted Impact of SDS as a Minor Component

**DOI:** 10.3390/pharmaceutics12030197

**Published:** 2020-02-25

**Authors:** Mahbubur Rahman, Stephanie Ahmad, James Tarabokija, Nathaniel Parker, Ecevit Bilgili

**Affiliations:** Otto H. York Department of Chemical and Materials Engineering, New Jersey Institute of Technology, Newark, NJ 07102, USA; mr485@njit.edu (M.R.); sa982@njit.edu (S.A.); jst35@njit.edu (J.T.); ndp46@njit.edu (N.P.)

**Keywords:** amorphous solid dispersions, spray drying, wettability, supersaturation, recrystallization, drug release

## Abstract

This study aimed to elucidate the impact of a common anionic surfactant, sodium dodecyl sulfate (SDS), along with hydroxypropyl cellulose (HPC) and Soluplus (Sol) on the release of griseofulvin (GF), a poorly soluble drug, from amorphous solid dispersions (ASDs). Solutions of 2.5% GF and 2.5%–12.5% HPC/Sol with 0.125% SDS/without SDS were prepared in acetone–water and spray-dried. The solid-state characterization of the ASDs suggests that GF–Sol had better miscibility and stronger interactions than GF–HPC and formed XRPD-amorphous GF, whereas HPC-based ASDs, especially the ones with a lower HPC loading, had crystalline GF. The dissolution tests show that without SDS, ASDs provided limited GF supersaturation (max. 250%) due to poor wettability of Sol-based ASDs and extensive GF recrystallization in HPC-based ASDs (max. 50%). Sol-based ASDs with SDS exhibited a dramatic increase in supersaturation (max. 570%), especially at a higher Sol loading, whereas HPC-based ASDs with SDS did not. SDS did not interfere with Sol’s ability to inhibit GF recrystallization, as confirmed by the precipitation from the supersaturated state and PLM imaging. The favorable use of SDS in a ternary ASD was attributed to both the wettability enhancement and its inability to promote GF recrystallization when used as a minor component along with Sol.

## 1. Introduction

In the last few decades, poor aqueous solubility of drugs, resulting in limited bioavailability, has appeared to be one of the major challenges in drug delivery systems [1]. Around 40% of the drugs available in the market and up to 90% of the new chemical entities in the development pipeline are poorly water-soluble [2]. Slow and incomplete dissolution of these drugs results in slow absorption in the aqueous environment of the gastrointestinal (GI) tract and eventually low bioavailability. To resolve these challenges, amorphous solid dispersions (ASDs) have been used as an effective platform approach [3,4]. In traditional ASDs, the drug is molecularly dispersed within a hydrophilic/amphiphilic polymeric matrix, resulting in a single-phase amorphous mixture, which inhibits recrystallization of the amorphous drug. As the kinetic solubility of the amorphous drug is higher than that of the crystalline drug, ASDs can enhance the dissolution rate–supersaturation generation significantly [5,6,7].

Although the higher free energy of the amorphous drug leads to apparent solubility levels of an order of magnitude higher than the crystalline counterpart of the drugs, this high energy also works as the driving force for spontaneous recrystallization from the solid state or supersaturated solution upon dissolution [8,9]. In conventional ASDs, an amorphous drug is molecularly mixed with a polymeric carrier, thus forming a binary system [10]. On the other hand, a significant improvement in the performance of drug ASDs has been reported with ternary systems, such as the drug–binary polymers [11] and drug–polymer–surfactant [12]. Several studies have suggested improvement in processability, dissolution performance, and storage stability owing to incorporation of a surfactant in ASDs over those without a surfactant [12,13,14,15,16,17]. Surfactants at high concentrations in ASDs have been used to increase the solubility of the drug (see, e.g., the references cited in Table 1). Moreover, the presence of a surfactant in the ASD formulation could increase wettability of the relatively hydrophobic drug [17,18], while it can also inhibit drug precipitation in the aqueous medium [19,20]. On the contrary, other studies [21,22,23,24] have suggested that surfactants negatively affected drug release from ASDs due to the competitive interaction between the drug–polymer–surfactant, resulting in drug recrystallization promotion from the supersaturated solutions. Therefore, to say the least, the impact and roles of surfactants in the dissolution of drug ASDs are complex and somewhat elusive, and the use of surfactants can be detrimental or beneficial depending on the specific drug–polymer–surfactant system and composition. Hence, a holistic investigation into the roles of surfactants in both the wettability enhancement and recrystallization inhibition of drugs along with polymers in ASDs is surely warranted.

A common anionic surfactant, sodium dodecyl sulfate (SDS), is typically used as a minor component, at 0.5%–2.5% *w*/*w*, in immediate release tablets or capsules [25]. On the other hand, when SDS has been used as a carrier along with polymers in ASDs; it has been used at much higher concentrations, typically at 10%–50% *w*/*w*, and/or as a major component of ASDs (see Table 1). This is not surprising as the use of surfactants and surfactants–polymers as carriers has been an emerging trend in the last two decades [26]. When polymers alone cannot achieve high kinetic solubilization, they are augmented with copious amounts of surfactants that can solubilize drugs through micellar solubilization and surfactant–polymer complex formation [22,27,28]. 

Even though anionic surfactants were reported to enhance wettability in most ASD studies, a cursory look at Table 1 reveals that wettability enhancement either by anionic surfactants like SDS alone or by anionic surfactants along with polymers has been rarely examined and quantified, especially in relation to the drug release from ASDs. As an exception, Lu et al. [18] investigated the impact of SDS in the wettability enhancement of simvastatin (SV) and its relation to the dissolution rate of the SV–polyvinyl pyrrolidone (PVP) solid dispersion via separate measurements of contact angle and water absorption into a packed powder bed. A similar method was used by Rahman et al. [29] to examine the wettability enhancement by SDS in hybrid nanoparticle–amorphous solid dispersions (HyNASDs) of griseofulvin (GF). Although both studies quantified the positive impact of SDS inclusion on drug wettability and drug release, Lu et al. [18] did not investigate the impact of SDS on the supersaturation maintenance–recrystallization kinetics, and Rahman et al. [29] spray-dried aqueous GF nanosuspensions to prepare HyNASDs and characterize them; however, GF solutions were not spray-dried to prepare ASDs, which is the focus of the current study.

Table 1 also shows that the effects of SDS on drug supersaturation maintenance have not been routinely examined in separate desupersaturation–recrystallization kinetic studies, unlike in recent studies such as Chen et al. [35], Deshpande et al. [24], and Feng et al. [17]. Moreover, several studies have indicated that SDS had a deleterious impact on drug supersaturation maintenance as it promoted drug recrystallization in the presence of polyvinyl pyrrolidone-vinyl acetate (PVP-VA) [22] and crystal growth in the presence of PVP [38]. It is likely that the impact of SDS in a ternary ASD is specific to the drug–polymer–SDS formulation and dependent on the surfactant concentration. As SDS was intended to be a carrier/solubilizer in most of the ASD studies, its loading was typically higher than 2.5% *w*/*w*, except in a few studies [18,32,36] that did not investigate the impact of SDS on drug wettability and/or recrystallization kinetics. In other words, in ASDs, SDS has not been commonly used as a minor component, i.e., 0.5%–2.5% *w*/*w*, unlike in typical immediate release dosages. In view of the existing literature, it is fair to state that the impact of SDS, as a minor component of ASDs, on both drug wettability enhancement and drug crystallization inhibition/supersaturation maintenance in the presence of polymers, specifically hydroxypropyl cellulose (HPC) and Soluplus (Sol), has not been systematically examined. It is hypothesized that the use of SDS as a minor component along with a drug-miscible polymer, which can provide solubilization and supersaturation maintenance, could boost supersaturation from the ASDs via wettability enhancement, without having any deleterious effect on drug recrystallization. Another driver for the use of SDS as a minor component of ASDs is that the anionic surfactants could be toxic and may cause gastrointestinal tract irritation [39,40], especially if used at high concentrations in ASDs with high drug doses. 

Unlike all previous studies, here we aimed to examine the impact of a common anionic surfactant, SDS, as a minor component of a drug–polymer–SDS ternary ASD on in vitro drug release while elucidating its roles in both wettability enhancement and recrystallization inhibition/promotion along with HPC/Sol. To this end, HPC (hydrophilic, partially miscible with GF) and Sol (amphiphilic, miscible with GF) were used as the matrix formers in the ASDs. A total of 2.5% griseofulvin (GF, a model poorly soluble drug) and HPC/Sol with various mass ratios of GF:polymer, along with or without SDS, were dissolved in acetone–water and spray-dried to prepare the ASDs. X-ray powder diffraction (XRPD), differential scanning calorimetry (DSC), and Raman spectroscopy were used to characterize any changes to GF crystallinity after spray drying and examine ASD formation. Release of GF from the spray-dried particles was studied using a standard dissolution tester along with UV-spectroscopy. Polarized light microscopy (PLM) was used to examine formation of GF crystals from a loose compact of ASD upon water addition. GF recrystallization inhibition/promotion upon use of the polymers and SDS was examined in the desupersaturation experiments via the solvent-shift method. We used the modified Washburn method to investigate the wettability enhancement of the drug by the polymers and SDS. The analysis of these experiments will allow us to (i) examine if the use of SDS along with a drug-miscible polymer, which can provide solubilization and supersaturation maintenance, could boost supersaturation from the ASDs; and (ii) determine how wettability enhancement, supersaturation maintenance/recrystallization kinetics, and drug solubilization can be affected by the use of SDS as a minor component.

## 2. Materials and Methods 

### 2.1. Materials 

Griseofulvin (GF) was selected as a model drug as it has a low solubility in water: ~8.9 mg/L at 25 °C. GF is considered a challenging drug as it crystallizes fast [41]. It was purchased from Letco Medical (BP/EP micronized grade, Decatur, AL, USA). Its melting point temperature *T*_m_ and glass transition temperature *T*_g_ are 220 °C and 89 °C [42]. Hydroxypropyl cellulose (HPC), which is a semi-crystalline polymer with low crystallinity and amorphous domains of sub-ambient *T*_g_ [43], was donated by Nisso America Inc. (SSL grade, New York, NY, USA). Soluplus^®^ (Sol), donated by BASF, is an amphiphilic graft copolymer made of polyvinyl caprolactam–polyvinyl acetate–polyethylene glycol. It has a *T*_g_ of 73 ± 2 °C [44]. Sodium dodecyl sulfate (SDS), a commonly used anionic surfactant, was purchased from GFS Chemicals, Inc. (Columbus, OH). Acetone (ACS reagent, ≥99.5%, BDH Analytical chemicals, Radnor, PA, USA) was used as an organic solvent.

### 2.2. Preparation of Spray-Dried Powders

Table 2 presents the formulations of various 2.5% *w/v* GF solutions with HPC/Sol/SDS in a mixture of 200 mL acetone–40 mL water that were prepared using a magnetic stirrer. As SDS is not soluble in acetone, water was used along with acetone to ensure dissolution of all the solid components. The solutions were sonicated for 30 min before feeding to the spray dryer. Sol and HPC were selected as they have a different hydrophilicity and glass transition temperature. GF:polymer mass ratios from 1:1 to 1:5 were selected to examine the impact of polymer loading on the ASD formation and GF release. The rationale for selecting 0.125% *w/v* SDS is as follows: when spray-dried, the powders with 1:1, 1:3, and 1:5 GF:polymer ratios are estimated to have 2.44%, 1.23%, and 0.83% *w*/*w* SDS, respectively, which are all in the range of 0.5%–2.5% *w*/*w* used in typical immediate release dosages. Moreover, when fully dissolved, the powders will provide 0.0005% *w/v* SDS in the dissolution medium, i.e., deionized water, which is well below the critical micelle concentration of SDS, i.e., 8 mM, 0.23% *w/v* [45]. Hence, the micellar solubilization of GF by SDS in the dissolution medium is purposefully minimized. A Procept 4M8-Trix spray dryer (Zelzate, Belgium) with a bi-fluid nozzle was used to dry 200 g GF solutions that were fed at 2.0 g/min. Drying air at 75 °C was fed at 0.27–0.30 m^3^/min. Parameters regarding atomization of the feed were adopted from Azad et al. [46]. After spray drying, a vacuum-desiccator was used to store dried particles in double plastic bags at room temperature before their characterization.

### 2.3. Characterization Methods

#### 2.3.1. Particle Sizing, Microscopy, and Solid-State Characterization

A Rodos/Helos laser diffraction system (Sympatec, NJ, USA) was used to measure the particle size of the as-received GF and spray-dried samples, based on Fraunhofer theory, following the method in Li et al. [47]. To examine the morphology of the spray-dried particles, samples were placed on a glass slide and observed under a polarized light microscope (PLM, Axio Scope.A1, Carl Zeiss Microscopy GmbH, Göttingen, Germany). An XRPD (PANanalytical, Westborough, MA, USA), equipped with Cu K_α_ radiation (λ = 1.5406 Å), was used to investigate the crystalline nature of the as-received GF, polymers (HPC/Sol), SDS, spray-dried samples, and physical mixtures (PMs). The PMs have the same formulations as those presented in Table 2, but prepared via blending. All samples were scanned within the range of 5° to 40° at a rate of 0.165 s^−1^ for the 2*θ* scanning mode. To estimate the % crystallinity of the spray-dried powders, HighScore Plus software was used following the method in Rahman et al. [48].

A Mettler-Toledo polymer analyzer (PolyDSC, Columbus, OH, USA) was used to perform DSC of the as-received GF, Sol, HPC, spray-dried samples, and physical mixtures (PMs) (see Section S1 of Supplementary Material for more details). As-received GF and PMs were heated from 25 °C to 250 °C at a rate of 10 °C/min under nitrogen gas flow. Spray-dried samples were heated from 25 °C to 70 °C and was held at 70 °C for 2 min to remove any residual solvent, then cooled back to 25 °C. In the last step, the samples were heated again at a rate of 10 °C/min from 25 °C to 250 °C. To assess the residual moisture/solvent content in the spray-dried samples, thermogravimetric analysis (TGA) was performed using a TGA/DSC1/SF Stare system (Mettler Toledo, Inc., Columbus, OH). Each sample weighing ~6.0–7.0 mg was placed in a ceramic crucible and heated at a rate of 10 °C/min under nitrogen flow from 25 °C to 150 °C.

A Fergie Imaging Spectrometer System (Princeton Instruments, Trenton, NJ, USA) with a 500-mW external diode laser processing at 785 nm wavelength was used to conduct Raman spectroscopy. Data acquisition time was 15 s per scanned spectrum (100–1800 cm^−1^) and each spectrum obtained was averaged over two scans. In this study, the data for the range of 1550–1800 cm^−1^ wavenumber was presented.

#### 2.3.2. Assay Testing and Drug Release from the Powders Prepared via Spray Drying

An assay testing was performed to determine drug content following the method in Li et al. [47]. A total of 100 mg of the sample powders was dissolved in 20 mL methanol under 30 min of sonication, followed by overnight storage to ensure complete solubilization of the GF particles. An aliquot of 100 µL was taken from the GF solution and diluted up to 10 mL using methanol. Using a UV spectrophotometer (Agilent, Santa Clara, CA, USA) at 292 nm wavelength, the absorbance of the samples was measured, while a pre-established calibration curve was used to calculate the drug concentration. For each formulation, mean drug content and the relative standard deviation (RSD) were calculated (*n* = 6). A Distek 2100C USP II (paddle apparatus) dissolution tester (North Brunswick, NJ, USA) was used to determine GF release from the powders prepared by spray drying and the physical mixtures (PMs) with 100 mg GF in 1000 mL deionized water at 37 °C stirred at 50 rpm paddle speed [48]. The powders were poured into the dissolution medium and deionized water from the dissolution vessels were spewed on top of any floating powder (only for Sol-based ASD samples without SDS) for the first 2 min to ensure complete submersion of the powders. The 4 mL samples were taken out manually at various intervals up to 210 min. The aliquots were filtered with a 0.1 µm PVDF membrane-type syringe filter before UV-spectroscopy measurements to minimize any confounding effect of the undissolved drug. The rationale for selecting deionized water is that it provided good discrimination among dissolution profiles of different GF formulations (nanocomposites–ASDs) in the previous studies [49,50,51]. Using UV–vis spectroscopy, the absorbance of the dissolved GF was measured at 296 nm wavelength. The concentration of the GF released was determined from a pre-established calibration curve. Six samples were tested for each formulation. Unless otherwise indicated, the relative % supersaturation is calculated based on GF concentration at 210 min and thermodynamic solubility of as-received GF at 37 °C.

#### 2.3.3. GF Wettability by Aqueous Polymer (Sol/HPC) Solutions with or w/o SDS

Wettability of GF was investigated using the modified Washburn method [52,53]. The penetration rate of aqueous polymer/SDS solutions into a packed bed of GF powder inside a cylindrical column was analyzed. The mass of liquid penetrating the GF powder bed was measured as a function of time using an Attension Sigma 700 (Biolin Scientific, Linthicum, MD, USA). The experimental method was adapted from Bilgili et al. [54] and Li et al. [51], and full details are provided in Section S2 of the Supplementary Material. The powder refers to as-received GF powder and the liquids are aqueous solutions of 15% Sol/HPC, 15% Sol/HPC with 0.125% SDS, and 0.125% SDS. The aqueous solutions and deionized water were saturated with griseofulvin (GF) and stirred overnight. Surface tension was measured using the Attension Sigma 700, and the apparent shear viscosity was measured with an R/S Plus Rheometer (Brookfield Engineering, Middleboro, MA, USA). The viscosity of water and the SDS solution were taken from Korson et al. [55] and Kushner et al. [56], respectively. The wetting effectiveness factor cos*θ*_s_/cos*θ*_w_ was estimated from the modified Washburn equation. Here, *θ*_s_ refers to the contact angle between the GF powder and the aqueous polymer/SDS solution and *θ*_w_ indicates the contact angle between the GF powder and the deionized water. The wetting effectiveness factor gauges the extent to which wettability of a drug is enhanced when the polymers (HPC/Sol) and SDS are used as solutes in deionized water [54]. 

#### 2.3.4. Impact of the Polymers and SDS on GF Recrystallization from Supersaturated Solutions

A solvent-shift method was used to examine the impact of polymer/surfactant on the potential depletion of GF supersaturation (desupersaturation) from a pre-supersaturated GF solution, which will help to elucidate drug recrystallization in the dissolution tests. To generate supersaturation, a concentrated solution of GF (100 mg GF dissolved in 20 mL acetone) was mixed with a pre-dissolved HPC/Sol aqueous solution in the dissolution tester having concentrations of 100, 300, and 500 µg/mL, emulating 1:1, 1:3, and 1:5 GF:polymer ratios in the powders, respectively, with 0.0005%/0.125% SDS or without SDS and separate aqueous solutions of 0.0005% and 0.125% SDS. Upon mixing, GF concentration rose to 76 to 99 µg/mL within few minutes. Measuring GF concentration over 210 min allowed us to determine how fast supersaturation is depleted due to recrystallization. These desupersaturation experiments were performed in triplicate. 

#### 2.3.5. Visualization of Solution-Mediated Recrystallization of GF

A loose compact of the spray-dried powders with S-HPC-1:5 and S-Sol-1:5, with and without SDS, was mounted onto a glass slide. After addition of about 20 µL of deionized water, the sample was observed under the polarized light microscope (PLM) and images were taken at 0, 1, 2, and 5 min.

## 3. Results and Discussion

We present and discuss various properties of the spray-dried powders (Section 3.1); the solid-state characterization of the drug in the powders and ASD formation (Section 3.2); in vitro drug release from the ASDs, and the effects of type and loading of the polymer and presence of SDS under supersaturating dissolution conditions (Section 3.3); and the roles of polymer–SDS in recrystallization inhibition/promotion (Section 3.4).

### 3.1. Properties of the Spray-Dried Powders

**Figure 1:**
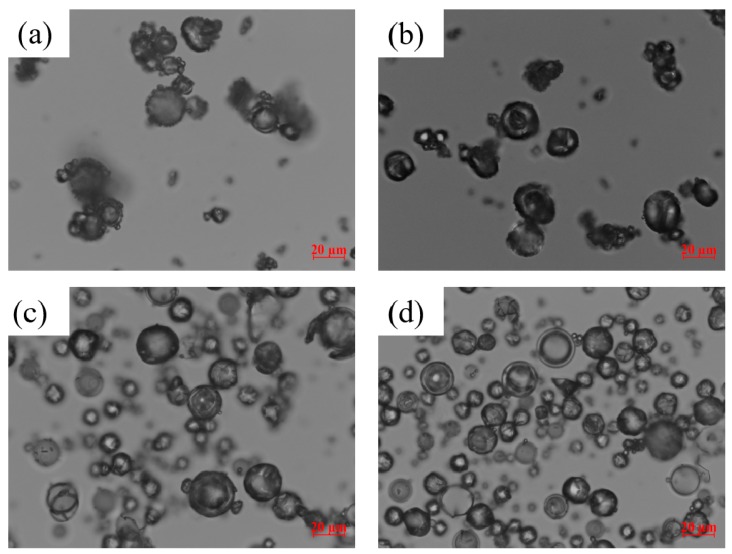
Light microscope images of the spray-dried particles prepared using the GF solution with a 1:3 GF:polymer mass ratio and 0.125% SDS/without SDS: (**a**) S-HPC-1:3, (**b**) S-HPC-1:3, SDS, (**c**) S-Sol-1:3, and (**d**) S-Sol-1:3, SDS. All images were taken at 50× magnification (scale bar: 20 µm).

Weight loss of 2.0% ± 0.3% was measured with TGA for all the samples, which confirmed that most of the solvents were removed during the spray drying. The powders with higher drug:polymer mass ratios had higher mean drug content (Table 3), while the RSD values ranged from 0.73%–4.45%, all below 6.0%, which suggests that the powders had acceptable content uniformity. Table 3 also shows that when the polymer concentration was increased, coarser particles were prepared because of the higher total solids loading in the more viscous feed [54,57,58]. Unlike this clear and strong effect of the polymer, the effect of SDS was relatively weak or its influence did not show a clear pattern. The spray-dried particles have rounded-donut shapes (see Figure 1).

### 3.2. Solid state Characterization of the Spray-Dried Powders

**Figure 2:**
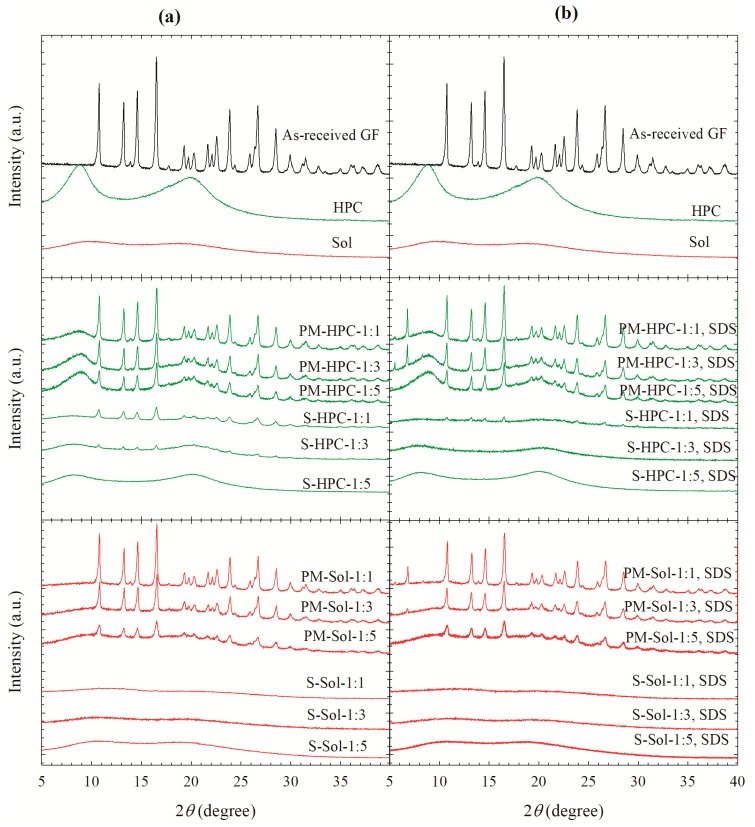
X-ray diffractograms of as-received GF, HPC, Sol, physical mixtures (PMs) of GF–HPC/Sol and the spray-dried powders prepared using the GF solutions with 1:1, 1:3, and 1:5 drug:polymer mass ratios: (**a**) without SDS and (**b**) with 0.125% SDS.

**Figure 3:**
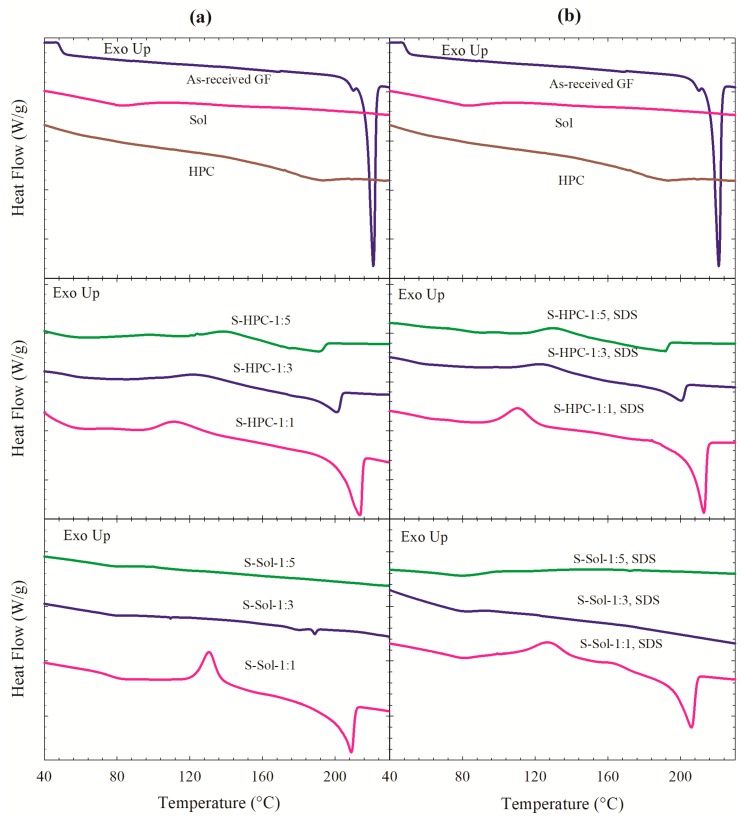
DSC traces of as-received GF, HPC, Sol, and the spray-dried powders prepared using the GF solutions with 1:1, 1:3, and 1:5 drug:polymer mass ratios: (**a**) without SDS and (**b**) with 0.125% SDS.

To examine any solid state changes to GF upon spray-drying, as-received GF, HPC, Sol, spray-dried powders, and corresponding physical mixtures (PMs) having identical formulations to those of the spray-dried powders were analyzed using XRPD (Figure 2) and DSC (Figure 3). Sharp peaks were observed for GF, whereas a halo pattern was observed for the polymers, suggesting their amorphous nature (Figure 2). Similar peaks at the same diffraction angles, but with lower intensity, were observed for the GF PMs prepared by blending, which can be explained by surface coverage and dilution of the GF crystals with the polymer. The peaks observed at 5.6°, 6.8°, and 8.3° in the diffractograms of physical mixtures (PMs) originate from SDS (see Section S3 of Supplementary Material; for proper scaling of GF peaks, the SDS diffractogram is excluded from Figure 2 and presented in Section S3).

In general, XRPD diffractograms of all Sol-based spray-dried powders with and without SDS, regardless of polymer loading, did not depict any diffraction peaks of GF. The halo patterns confirm the formation of amorphous solid dispersion (ASD). Small peaks were visible in the diffractograms of S-HPC-1:3, S-HPC-1:1, and S-HPC-1:1 SDS powders, which had 11.5%, 27.7%, and 6.5% crystallinity, respectively (Table 4). Despite being largely amorphous, strictly speaking, these powders should be referred to as solid dispersions; but, for the sake of simplicity, we call all powders ASDs while recognizing that some of them had notable crystalline content. The XRPD results overall suggest that (i) amorphous GF was molecularly dispersed by Sol matrix owing to their good miscibility even at a 1:1 mass ratio and whether SDS was present or not; (ii) GF was only partially miscible with HPC, and it required a high HPC loading (1:5 GF:HPC) to ensure formation of ASD—a lower HPC loading could not prevent GF recrystallization during or after spray drying; and (iii) SDS helped to disperse or solubilize GF in the HPC matrix, thus allowing ASD formation at a lower HPC loading (1:3 GF:HPC ratio). However, SDS could not prevent recrystallization when the GF:HPC mass ratio was 1:1. During spray drying, rapid evaporation of the solvents raised viscosity dramatically, resulting in kinetic arrest of the drug molecules by the polymers, thus forming ASD [59]. Therefore, besides drug–polymer miscibility, fast drying kinetics in the spray dryer played a role in the formation of ASD from solution-based (S) feeds, even for a partially miscible (GF–HPC) system.

As-received GF showed a fusion peak with *T*_m_ of 220.1 °C and fusion enthalpy Δ*H*_f_ of 101.8 J/g (Figure 3). Although HPC is XRPD amorphous, the event around 170–200 °C for HPC is likely due to the melting of the small crystalline domain of largely amorphous HPC [43]. The *T*_g_ of HPC, which is in the range of −25 to 0 °C [43], could not be measured due to limitation of our equipment. A glass transition for Sol (amorphous) was noted at 72.4 °C. A single *T*_g_ was observed corroborating the molecular level dispersion [60,61] in all powders except S-HPC-1:1 with 28% GF crystallinity (see Table 4). Only a glass transition was noted in the DSC traces of S-Sol-1:3, SDS; S-Sol-1:5, SDS; and S-Sol-1:5. DSC traces of other ASDs depicted a glass transition, an exothermic event likely due to the re-crystallization of the amorphous GF, and a subsequent endothermic event due to fusion of either the recrystallized GF or pre-existing GF crystals (Figure 3). Recrystallization of GF from ASDs, especially with lower polymer loading, may occur because the high temperatures above *T*_g_ during the DSC measurement render amorphous drug molecules mobile. The absence of recrystallization and higher temperature of the recrystallization transition *T*_rc_ (if it occurred at all) in the Sol-based powders compared to the HPC-based powders suggest better miscibility and stronger interactions between the Sol–GF molecules than the HPC–GF molecules. For HPC-based powders, *T*_rc_ increased (recrystallization delayed to higher temperature) and −Δ*H*_rc_ decreased at a higher HPC loading, which accords well with Wlodarski et al. [60]. Melting point depression was also observed in the DSC traces of the PMs (see Section S3), which is another indicator of drug–polymer miscibility [62,63].

Table 4 indicates that ASDs with higher polymer loading either did not have a melting point or exhibited a fusion event with a lower *T*_m_ and lower Δ*H*_f_ regardless of the presence/absence of SDS. One reason is the dilution of the drug and presence of a lower drug content at a higher polymer loading (refer to Table 3). However, ASDs had lower *T*_m_ and Δ*H*_f_, even if detected, as compared with the PMs of identical drug loading (refer to Table S1 of Supplementary Material). For the same polymer/SDS composition, the formulations with Sol had either no melting point or had a lower melting point, higher melting point depression, and lower Δ*H*_f_ than those with HPC. It is reasonable to attribute these observations to (i) stronger molecular interactions and better miscibility for GF–Sol than GF–HPC; (ii) higher *T*_g_ and more amorphous GF in the Sol-based powders than in the HPC-based powders; and (iii) a higher extent of drug–polymer interactions and solubilization in the ASDs and during the thermal treatment with DSC when the polymer loading was higher. At the same loading for a given polymer, the presence of SDS either led to disappearance of the melting point or slightly higher melting point depression, which suggests that SDS appears to help GF molecular dispersion or solubilization to a limited extent. These findings from DSC traces are largely in agreement with the findings from the XRPD diffractograms regarding the effects of GF–polymer miscibility, polymer loading, and presence of SDS on the GF’s solid state. 

**Figure 4:**
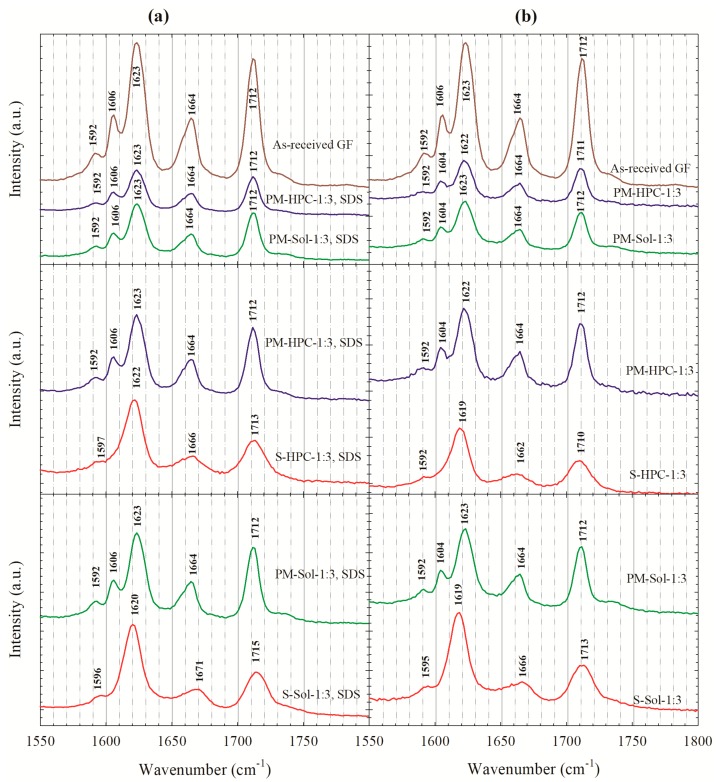
Raman spectra of as-received GF, physical mixtures (PMs) of GF–HPC/Sol at a 1:3 drug:polymer ratio, and the spray-dried powders prepared using the GF solution with a 1:3 GF:polymer mass ratio: (**a**) with 0.125% SDS and (**b**) without SDS.

Figure 4 illustrates the Raman spectra of as-received GF, the spray-dried formulation with a 1:3 GF:polymer ratio, and the corresponding PMs. The spectra of as-received GF and PMs of GF agree well with the Fourier transform Raman data of Feng et al. [64] and Raman data of Żarów et al. [65] for crystalline GF. In the Raman lines of the GF crystals, the spectral range from 1550 to 1800 cm^−1^ corresponds to the C=O stretching vibrational frequencies and displays a characteristic triplet of lines. For the crystalline GF, the first peak of the triplet splits into three additional peaks at 1592, 1606, and 1623 cm^−1^ resulting from the interaction between the two C=O stretching modes and partial conjugation in the neighboring cyclohexane ring [65]. In the spectra of the spray-dried powders, this splitting no longer exists, and the two broader peaks were observed which were shifted from 1592 to 1596 cm^−1^ and 1623 to 1620 cm^−1^, while the GF line at 1606 cm^−1^ disappeared. The second peak (1664 cm^−1^) corresponds to the stretching of the carbonyl group (C=O) of cyclohexane, and the third peak (1712 cm^−1^) corresponds to the stretching of carbonyl group (C=O) of the benzofuran [65]. The broadening and the change in the relative peak intensity within the band spectral window of 1550 to 1800 cm^−1^ correspond to the asymmetric vibration of the C=O of griseofulvin (H bond-acceptor), which might form hydrogen (H)-bonds with the –OH groups of Sol (H bond-donor). These differences provide evidence for the conversion from crystalline GF to amorphous GF and interactions between GF and polymers at the molecular level. While S-HPC-1:3 with and w/o SDS exhibited the disappearance of the GF line at 1606 cm^–1^ (Figure 4a,b), they exhibited subtler peak shifts than S-Sol-1:3 with and without SDS, which may signify greater interactions between GF–Sol than GF–HPC. 

The solubility parameter difference between a drug and polymer may be used to assess their miscibility theoretically. They are regarded as miscible, partially miscible, and immiscible for the differences below 7.0 MPa^1/2^, between 7.0 MPa^1/2^ and 10 MPa^1/2^, and above 10 MPa^1/2^, respectively [66,67]. For GF, HPC, and Sol, the parameters are 12.2 [68], 24.0 [69], and 19.4 MPa^1/2^ [70], respectively. GF–Sol (7.2 is MPa^1/2^) appears to be borderline miscible, whereas GF–HPC (11.8 MPa^1/2^) is immiscible. The solubility parameter prediction is reasonably accurate for Sol–GF as the thermal analysis, spectroscopy, and XRPD results suggest GF–Sol are miscible and molecularly interact stronger than GF–HPC. However, GF–HPC exhibits partial miscibility unlike what the solubility parameters of GF–HPC suggest. The theoretical models behind the solubility parameter predictions have various limitations, as discussed by other researchers [41,71]. Indeed, the absence of diffraction peaks in XRPD in several HPC-based powders and significant melting depression in DSC along with the Raman line shifts indicate the interaction of HPC with GF, leading to partial miscibility unlike what the solubility parameters predict.

### 3.3. Drug Release from the Powders Prepared by Spray Drying

**Figure 5:**
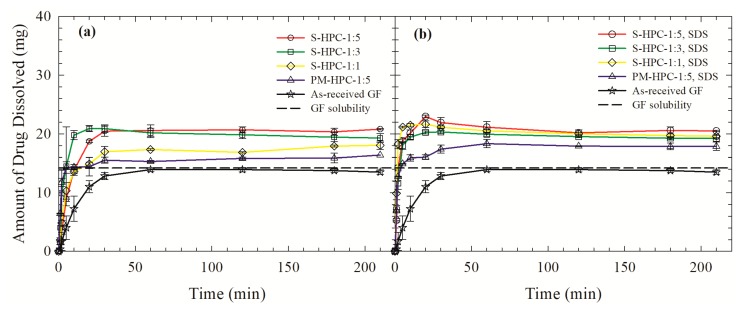
Evolution of drug release from as-received GF, physical mixture (PM) with a 1:5 GF:HPC mass ratio, and spray-dried powders prepared using the GF solutions with 1:1, 1:3, and 1:5 GF:HPC mass ratios: (**a**) HPC without SDS, (**b**) HPC with SDS.

**Figure 6:**
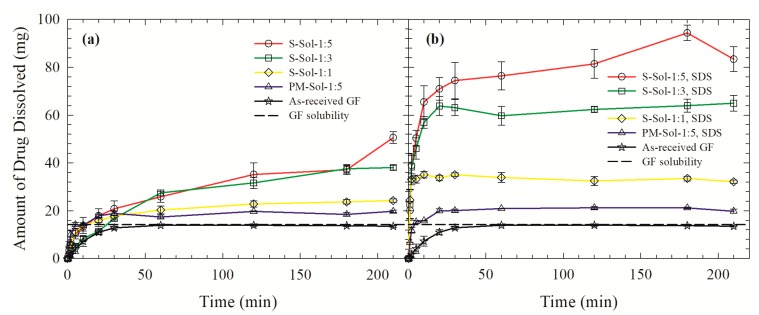
Evolution of drug release from as-received GF, physical mixture (PM) with 1:5 GF:Sol mass ratio, and spray-dried powders prepared using the GF solutions with 1:1, 1:3, and 1:5 GF:Sol mass ratios: (**a**) Sol without SDS, (**b**) Sol with SDS.

Figure 5 and Figure 6 illustrate the timewise evolution of GF release from the spray-dried powders and the PM with the highest polymer loading (1:5 GF:polymer). Samples with 100 mg GF dose were dissolved in 1000 mL deionized water at 37 °C. The GF release from the PMs, wherein GF was simply blended with HPC/Sol/SDS, was slightly faster than that from the as-received GF particles (*d*_10_: 3.04 ± 0.1 µm*, d*_50_: 10.0 ± 0.17 µm, and *d*_90_: 28.2 ± 0.14 µm) and attained a higher plateau value. The higher GF solubility of the crystalline GF particles in the presence of HPC/Sol/SDS could be one reason for the observed faster release from the PM: 14.2 mg/L, 18.6 ± 0.2 mg/L, and 17.6 ± 0.3 mg/L in deionized water, aqueous medium corresponding to full dissolution of Sol–SDS (1:5 GF:Sol ratio), and that corresponding to a full dissolution of HPC–SDS (1:5 GF:HPC ratio), respectively, at 37 °C. Another explanation is the wettability enhancement of the hydrophobic drug in the presence of a polymer or surfactant, which is scrutinized below. Owing to the wettability enhancement, it is likely that GF aggregates in the as-received drug powder were well-dispersed in the dissolution medium [51].

**Figure 7:**
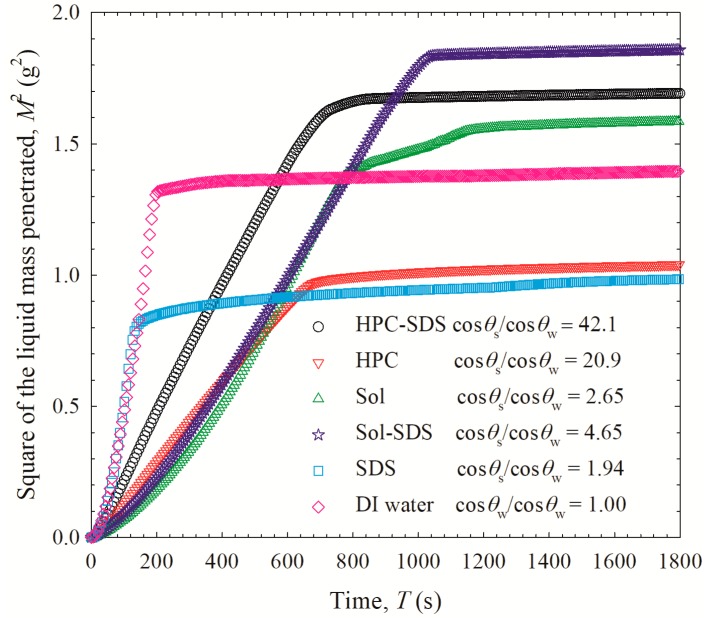
Temporal evolution of the liquid mass M penetrating a packed bed of as-received GF particles. Liquids: GF-saturated deionized (DI) water and various GF-saturated aqueous solutions of 15% HPC/Sol with 0.125% SDS and without SDS, and 0.125% SDS alone. The legend shows the wetting effectiveness factor cos*θ*_s_/cos*θ*_w_ calculated for each liquid. As deionized water was taken as the reference, cos*θ*_s_/cos*θ*_w_ equals 1, by definition, for water.

The wettability enhancement of GF was examined by investigating the penetration of aqueous polymer/SDS solutions into the GF beds. Figure 7 illustrates the evolution of the liquid mass (*M*) penetration in time (*T*). The slope of the modified Washburn equation was obtained from the slope of the linear region of the *M^2^–T* data (see Section S2 and Table S2 of Supplementary Material for full details). The initial ~20 s was not considered due to transient behavior; data points that deviated from the linear region were also excluded. The modified Washburn equation fitted the *M^2^–T* data in Figure 7 well (*R^2^* ≥ 0.98). The polymers/surfactant enhanced the wettability of the hydrophobic drug, as signified by the higher wetting effectiveness factor (see cos*θ*_s_/cos*θ*_w_ values in Figure 7). 

GF was released faster and/or to a higher extent from the spray-dried powders than the PMs, which can be attributed to the presence of amorphous GF in the former that has a higher apparent solubility than crystalline GF in the PM (Figure 5 and Figure 6). The poor wettability of the S-Sol formulation hindered the dissolution of Sol and erosion of the spray-dried particles, which in turn retarded the GF release (Figure 6a). Inclusion of SDS in the ASDs resulted in faster GF release, but the increase was more notable for the Sol-based ASDs than the HPC-based ASDs. The analysis of the wetting effectiveness factors obtained from the modified Washburn method (refer to Figure 7) shed some light on this observation. Presence of SDS has almost doubled the wetting effectiveness of the dissolved polymer in water and the rank order of wettability enhancement is HPC–SDS > HPC > Sol–SDS > Sol, which is in accordance with the hydrophilic nature of HPC and amphiphilic nature of Sol. Hence, poor wettability of Sol-based ASDs was mitigated upon incorporation of SDS as a minor component, which led to significant dissolution improvement. On the other hand, HPC-based ASDs did not significantly benefit from the incorporation of SDS as HPC is more hydrophilic than Sol and significant GF recrystallization occurred, which will be further examined below. 

Figure 5 shows that only slight supersaturation (max. ~50%) was achieved fast upon dissolution of HPC-based ASDs. An increase in polymer loading led to slight increase in the supersaturation attained. Similarly, the presence of SDS only increased supersaturation for the lowest HPC S-HPC-1:1; at higher HPC loadings, the effect of SDS was not notable. A cursory look at Figure 5 and Figure 6 suggests that Sol-based ASDs exhibited higher GF supersaturation than HPC-based ASDs and that inclusion of SDS as a minor component of ASD had a drastic impact on the attainment of high supersaturation fast: e.g., 430% supersaturation at 30 min for S-Sol-1:5, SDS. In fact, this ASD maintained supersaturation way above 430% for an additional 180 min and attained 570% supersaturation at 180 min. It is suggested that during the intestinal transit time (>180 min), maintaining a drug in the amorphous form, without precipitation, would often be sufficient for achieving the necessary bioavailability [72]. A higher polymer loading achieved a higher GF supersaturation, especially for Sol-based GF ASDs with SDS.

**Figure 8:**
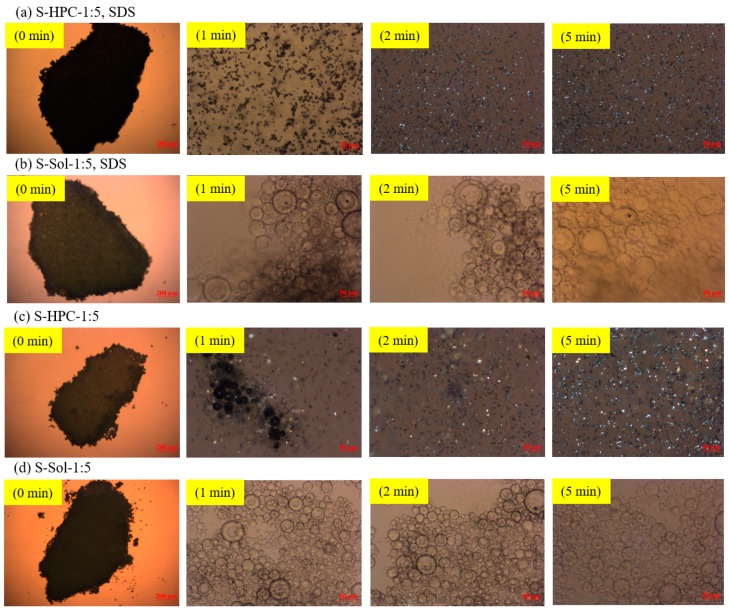
PLM images of a loose compact of the spray-dried particles with a 1:5 drug:polymer mass ratio in 20 µL deionized water: (**a**) HPC with SDS, (**b**) Sol with SDS, (**c**) HPC without SDS, and (**d**) Sol without SDS. The images were taken at 0 (before adding water), 1, 2, and 5 min after the addition of deionized water. Except for the 0 min image (5× magnification, scale bar: 200 µm), which focused on the compact, all other images focused on particles that emanated from the surface of the compact, which were captured at 20× magnification (scale bar: 50 µm).

The high GF supersaturation achieved by Sol-based ASDs as compared with HPC-based ASDs can be attributed to greater GF–Sol miscibility and interactions, a higher GF solubilization in Sol micelles, which increased with a higher Sol loading, and a stronger GF recrystallization inhibition imparted by Sol than by HPC, which will be examined in the next section. To make the GF recrystallization more lucid, let us examine the PLM images (Figure 8) of loose ASD compacts imbibed with a 20 µL deionized water droplet. The addition of water to the S-HPC-1:5, SDS compact (Figure 8a), and S-HPC-1:5 compact (Figure 8c) resulted in immediate dissolution of the compact and recrystallization of amorphous GF (see the shiny crystals in the respective images). On the other hand, for the Sol-based compact with SDS (Figure 8b) and without SDS (Figure 8d), the swollen ASD particles eroded from the compact gradually, and released amorphous GF from the Sol matrix. The images show spherical, swollen ASD particles. The amorphous GF in these ASDs did not recrystallize after 5 min, which implies that the phase separation did not occur and the undissolved ASD powders stayed in the amorphous form and facilitated the supersaturation generation of GF (Figure 8b,d). Note that when ASDs contact and absorb water in the dissolution medium, the amorphous drug may phase-separate and precipitate [73,74]. Water acts as a plasticizer and enhances the mobility of the drug molecules by reducing the *T*_g_ of the ASD [74]. As the HPC-based ASDs have an already lower *T*_g_ than the Sol-based ASDs (refer to Table 4), the plasticizing effect of water seems to have a more pronounced impact on the HPC-based ASDs.

### 3.4. On the Roles of the Polymer/SDS in Recrystallization Inhibition/Promotion

**Figure 9:**
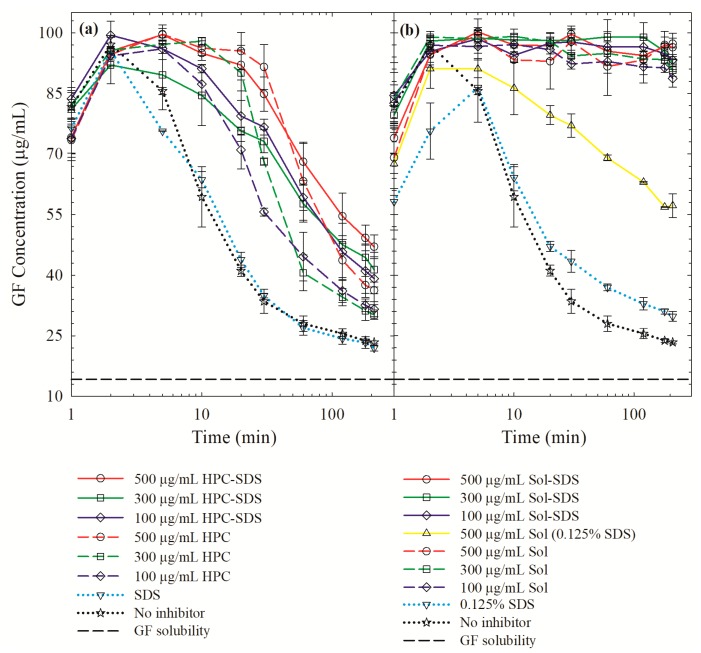
GF desupersaturation curves for a supersaturated 20 mL GF–acetone solution mixed with 1000 mL aqueous solutions of 500 µg/mL, 300 µg/mL, and 100 µg/mL of HPC/Sol–SDS and HPC/Sol (corresponding to 1:5, 1:3, and 1:1 drug:polymer formulations), SDS only, and deionized water without any recrystallization inhibitor: (**a**) HPC and (**b**) Sol. Unless otherwise indicated, 0.0005% *w/v* (5 µg/mL) SDS was used for the formulations with SDS. For aqueous 500 µg/mL Sol–SDS solutions, SDS was used at 0.0005% *w/v* and 0.125% *w*/*v*. The initial concentration of GF right after mixing was targeted at 100 µg/mL.

To gain further insights into the roles of the polymer/SDS in recrystallization inhibition/promotion, desupersaturation experiments were performed via the solvent-shift method. The addition of a GF–acetone solution to the aqueous polymer/SDS solution led to a supersaturation spike; 76–99 µg/mL GF dissolved within 2 min (see Figure 9). In the absence of additives, GF started to precipitate from the supersaturated solution immediately, which accords well with GF’s well-known fast crystallizing nature [41]. HPC with or w/o SDS could not prevent depletion of supersaturation (desupersaturation). In fact, SDS appears to promote the nucleation, especially at 0.125% in the solution but led to slower GF depletion later. The impact of SDS was insignificant at 0.0005%, which corresponds to the SDS concentration that would be generated by full dissolution of the ASDs with SDS. Finally, Sol was able to maintain high GF supersaturation for at least 180 min with a small drop at 210 min, signifying its superior GF crystallization inhibition. The presence of SDS and a higher Sol led to a slightly higher supersaturation; but these differences are small and within the experimental errors. In the presence of 0.125% SDS alone or with 500 µg/mL Sol in the dissolution medium, GF nucleation, and recrystallization occurred (see Figure 9b), albeit to a smaller extent than for GF alone. This finding confirms that a high concentration of SDS could indeed promote nucleation/recrystallization from the supersaturated GF solution. SDS promoted drug recrystallization in the presence of PVP-VA [22] and crystal growth in the presence of PVP [38]. In summary, no adverse impact of SDS on GF recrystallization was observed when it was used as a minor component along with Sol.

**Figure 10:**
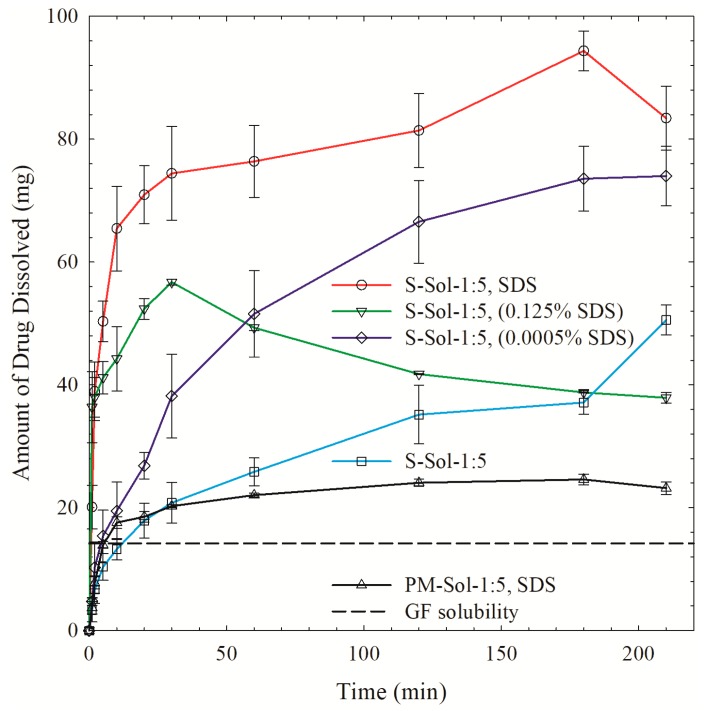
Evolution of GF release from a physical mixture (PM) with Sol–SDS and spray-dried powders prepared using the GF solution with a 1:5 GF:Sol mass ratio and with/without SDS in the formulation. Deionized water was used as the dissolution medium for the formulation with SDS (S-Sol-1:5, SDS) and the physical mixture. For the formulation without SDS (S-Sol-1:5), an aqueous solution of 0.125% *w/v* SDS, aqueous solution of 0.0005% *w/v* SDS, and deionized water were used as dissolution media.

To further understand the effect of SDS in Sol-based ASDs, S-Sol-1:5 (without SDS) was dissolved in 1000 mL of deionized water, an aqueous solution of 0.0005% SDS, and an aqueous solution of 0.125% SDS. In these cases, SDS was introduced to ASDs externally, outside the ASD particles. In S-Sol-1:5, SDS; the SDS was in the ASD particles, whose dissolution in water would yield a 0.0005% SDS. Figure 10 shows that the external addition of 0.0005% SDS significantly improved the GF release from S-Sol-1:5, which corroborates the wettability enhancement mechanism. Adding 0.125% SDS led to even faster supersaturation due to faster wettability; however, the released GF recrystallized, which is in line with the desupersaturation test (Figure 9b). When too much surfactant is used either in the ASD or in the dissolution medium, SDS molecules could compete with drug molecules to interact with the polymer (Sol) molecules, which in turn interferes with the crystallization, inhibiting capability of the polymer (Sol), resulting in recrystallization [22]. Liu et al. [22] reported a similar finding for sorafenib–PVP-VA ASD. Although the SDS–PVP-VA combination enhanced the solubility of sorafenib, the competitive interactions of SDS and sorafenib with the VA group of PVP-VA, which is responsible for maintaining supersaturation of sorafenib, resulted in a reduced ability of PVP-VA to maintain a high extent of sorafenib supersaturation during the dissolution. These findings signify the criticality of the SDS concentration in ASDs and the complexity of the SDS impact on drug release. 

Here, the fastest supersaturation occurred when SDS was internally added or incorporated into the ASD (S-Sol-1:5, SDS; dissolution in deionized water). It is likely that the presence of SDS in the ASD particles led to faster wettability enhancement as the GF is already available at the surface, locally obviating the need for SDS molecules adsorbing onto ASD microparticles from the dissolution medium. The higher local SDS concentration in the ASD particle and its boundary layer will also facilitate water imbibition into the Sol matrix and its faster erosion, leading to faster release of GF. Interestingly, despite exhibiting a much slower build-up of supersaturation, S-Sol-1:5 ASD (without SDS) tended to a plateau supersaturation at 210 min in a 0.0005% SDS solution, which is slightly below the supersaturation achieved by S-Sol-1:5, SDS in deionized water. This finding suggests that having the SDS internally as a minor component of the ASD along with GF–Sol led to a slightly higher kinetic solubility of GF compared to its external addition. Overall, our findings suggest that the GF release from the Sol-based ASDs was drastically improved owing to the GF wettability enhancement by SDS provided that SDS is a minor component with an effective GF recrystallization inhibitor present Sol as the major component in the ternary ASD.

## 4. Conclusions and Outlook

While SDS has been commonly used as a solubilizer/carrier typically at 10%–50% in many ternary ASDs, its use as a minor component (<2.5% *w*/*w*) in GF ASDs has been investigated in this study with a focus on the elucidation of its impact on GF release through wettability enhancement and solution-mediated GF recrystallization. Indeed, SDS was shown to promote GF recrystallization and could lead to severe GF recrystallization during the dissolution if used at a high concentration. The favorable use of SDS for enhancing GF release from a ternary GF–Sol–SDS ASD was attributed to both the wettability enhancement and the SDS’s inability to promote GF recrystallization when used as a minor component along with Sol, which provides significant solubilization of the drug (GF) and inhibits GF recrystallization alone. Higher Sol loading in the ternary ASDs resulted in higher supersaturation. The ASD with 1:5:0.05 GF:Sol:SDS composition, even with 0.83% SDS *w*/*w*, achieved 430% GF supersaturation within 30 min and ~500% after 210 min. The use of SDS as a minor component here significantly improved the wettability of GF–Sol, without having any deleterious impact on GF recrystallization, which is a common problem in polymer–surfactant carrier systems. The use of such low concentrations of SDS even in high-dose applications of ASDs also alleviates any concern associated with the toxicity of anionic surfactants. The high GF supersaturation was only possible due to the highly favorable properties of Sol because very limited supersaturation was achieved by HPC-based ASDs with or without SDS. Sol-based ASDs have a higher *T*_g_ owing to a higher *T*_g_ of Sol than HPC-SSL; Sol has greater miscibility and stronger molecular interactions with GF than HPC, as revealed by XRPD, DSC, and Raman spectroscopy analysis; and it inhibits GF recrystallization effectively, as suggested by the solvent-shift experiments. The only drawback of Sol was its amphiphilic nature and ensuing poor wettability of the Sol-based ASDs that contain a hydrophobic drug. Adding SDS as a minor component alleviated this problem, which enabled fast supersaturation from Sol-based ASDs. A future study will entail examining the stability of these ASD formulations and optimizing the SDS concentration for various high drug doses. The generality of the use of anionic surfactants as a minor component will also be tested with other drug–polymer–surfactants to assess the supersaturation generation–maintenance benefits.

## Figures and Tables

**Table 1:** Formulation composition of drug amorphous solid dispersion (ASDs) with sodium dodecyl sulfate (SDS) in various studies and survey of the use of wettability and desupersaturation tests.

Drug	Drug Loading (% *w*/*w*)	Polymer ^a^	Drug:Polymer:SDS	Wettability Testing	DeS Testing ^b^	References
Ketoprofen	10%	PEG	1:8:1	No	No	Mura et al. [19]
Tacrolimus	10%	CMC-Na	1:8:1	No	No	Park et al. [30]
Docetaxel	5%–9%	PVP K30	1:9:1–1:19:1	No	No	Moes et al. [31]
Valsartan	50%–67%	HPMC	1:0.42:0.08–1:0.67:0.33	No	No	Yan et al. [32]
Sulfathiazole	33%–50%	PVP K29/32	1:1:0.1–1:1:1	No	No	Dave et al. [33]
Simvastatin	20%–33%	PVP K29/32	1:3:0.02–1:3:0.06	Yes	No	Lu et al. [18]
Sorafenib	20%–50%	Soluplus	1:0.9:0.1–1:4.5:0.5	No	No	Truong et al. [34]
Tacrolimus	20%–33%	HPMC	1:1:1–1:1:3	No	No	Jung et al. [27]
Felodipine	23%	Soluplus	1:3:0.2–1:3:0.4	No	Yes	Chen et al. [35]
Itraconazole	50%	Soluplus, PVP VA64	1:0.5:0.5	No	Yes	Deshpande et al. [24]
Itraconazole	20%	HPMC-AS	1:3.75:0.25–1:2.75:1.25	No	Yes	Feng et al. [17]
Sirolimus	16%–48%	HPMC	1:1:0.05–1:5:0.1	No	No	Kim et al. [36]
Nifedipine	14%–40%	Kolliphor, Soluplus	1:1:0.5–1:4:2	No	No	Muralichand and Bhikshapathi [37]

^a^ CMC-Na: Carboxymethylcellulose-sodium, HPMC: hydroxypropyl methylcellulose, HPMC-AS: hydroxypropyl methylcellulose-acetyl succinate, PEG: polyethylene. glycol, PVP: polyvinyl pyrrolidone, PVP-VA: polyvinyl pyrrolidone-vinyl acetate. ^b^ DeS testing: Desupersaturation testing.

**Table 2:** Formulations of the griseofulvin–hydroxypropyl cellulose/Soluplus (GF–HPC/Sol) solutions with or without SDS fed to the spray dryer.

Formulation ID ^a^	GF (% *w*/*v*) ^b^	Polymers (% *w*/*v*) ^b^	SDS (% *w*/*v*) ^b^
S-Sol-1:5	2.5	12.5	0
S-Sol-1:3	2.5	7.5	0
S-Sol-1:1	2.5	2.5	0
S-Sol-1:5, SDS	2.5	12.5	0.125
S-Sol-1:3, SDS	2.5	7.5	0.125
S-Sol-1:1, SDS	2.5	2.5	0.125
S-HPC-1:5	2.5	12.5	0
S-HPC-1:3	2.5	7.5	0
S-HPC-1:1	2.5	2.5	0
S-HPC-1:5, SDS	2.5	12.5	0.125
S-HPC-1:3, SDS	2.5	7.5	0.125
S-HPC-1:1, SDS	2.5	2.5	0.125

^a^ S denotes solution-based feed; the ratios refer to the mass of the GF and polymer. ^b^ % *w/v* w.r.t. 200 mL acetone–40 mL deionized water mixture.

**Table 3:** Particle sizes of the powders prepared via spray drying and their drug content.

Formulation ID	Characteristic Sizes from the Volume-Based Size Distribution (µm)			GF Content, RSD (% *w*/*w*, %)
	*d*_10_ ± SD	*d*_50_ ± SD	*d*_90_ ± SD	
S-Sol-1:5	7.03 ± 0.2	18.3 ± 0.2	38.3 ± 0.1	14.8, 1.79
S-Sol-1:3	6.08 ± 0.1	14.3 ± 0.0	32.4 ± 0.1	22.1, 1.76
S-Sol-1:1	3.46 ± 0.2	10.9 ± 0.1	21.5 ± 0.0	44.8, 3.46
S-Sol-1:5, SDS	6.23 ± 0.1	20.8 ± 0.1	40.1 ± 0.2	14.6, 4.45
S-Sol-1:3, SDS	4.11 ± 0.0	12.3 ± 0.0	33.2 ± 0.1	21.5, 2.02
S-Sol-1:1, SDS	5.03 ± 0.1	11.0 ± 0.1	20.2 ± 0.0	42.3, 2.21
S-HPC-1:5	6.48 ± 0.2	21.5 ± 0.4	42.3 ± 0.2	15.0, 2.65
S-HPC-1:3	5.87 ± 0.1	15.4 ± 0.3	33.5 ± 0.1	24.0, 1.51
S-HPC-1:1	5.28 ± 0.1	12.7 ± 0.2	30.3 ± 1.2	44.9, 1.67
S-HPC-1:5, SDS	7.10 ± 0.2	22.6 ± 0.2	40.3 ± 0.3	15.1, 3.30
S-HPC-1:3, SDS	6.48 ± 0.0	15.8 ± 0.6	31.3 ± 1.0	24.4, 2.56
S-HPC-1:1, SDS	7.05 ± 0.2	13.0 ± 0.9	26.9 ± 0.8	42.7, 0.73

**Table 4:** Temperature–enthalpy values of various thermal events observed in the DSC traces and crystallinity estimated from the XRPD diffractograms.

Formulation ID ^a^	*T*_g_ (°C)	*T*_rc_ (°C)	Δ*H*_rc_ (J/g)	*T*_m_ (°C)	Δ*H*_f_ (J/g)	Crystallinity (%)
S-Sol-1:5	80.6	—	—	—	—	—
S-Sol-1:3	80.4	—	—	189	0.64	—
S-Sol-1:1	77.7	131	−14.1	209	23.2	—
S-Sol-1:5, SDS	77.4	—	—	—	—	—
S-Sol-1:3, SDS	80.0	—	—	—	—	—
S-Sol-1:1, SDS	74.6	127	−9.26	206	25.4	—
S-HPC-1:5	52.9	139	−3.35	192	6.64	—
S-HPC-1:3	53.2	122	−4.36	201	13.2	11.5
S-HPC-1:1	—	111	−9.70	213	34.8	27.7
S-HPC-1:5, SDS	51.7	130	−2.43	191	7.43	—
S-HPC-1:3, SDS	57.7	124	−3.86	200	11.6	—
S-HPC-1:1, SDS	58.9	109	−15.8	213	38.9	6.5

^a^ S denotes solution-based feed; the ratios refer to the mass of the GF and polymer.

## Supplementary Materials

The following are available online at https://www.mdpi.com/1999-4923/12/3/197/s1: Figure S1. DSC traces of the physical mixtures (PMs) of GF–HPC/Sol with 1:1, 1:3, and 1:5 drug:polymer mass ratios and SDS and without SDS: (**a**) PMs with Sol and (**b**) PMs with HPC; Table S1. Glass transition temperature, melting point temperature, and fusion enthalpy of the physical mixtures (PMs) obtained from DSC traces; Table S2. Properties of deionized water and aqueous polymer–SDS solutions as well as the wetting effectiveness factor determined using the modified Washburn method; and Figure S2. X-ray diffractograms of as-received GF and SDS powders.
