# Supplementary material for: Spray-Dried Amorphous Solid Dispersions of Griseofulvin in HPC/Soluplus/SDS: Elucidating the Multifaceted Impact of SDS as a Minor Component
Source: Pharmaceutics. 2020 Feb 25;12(3):197. doi: 10.3390/pharmaceutics12030197 (PMC7150901; doi:10.3390/pharmaceutics12030197)
Supplement: Supplementary file 1 [file pharmaceutics-12-00197-s001.pdf]

pharmaceutics

Supplementary Material: Spray-Dried Amorphous Solid Dispersions of Griseofulvin in HPC/Soluplus/SDS: Elucidating the Multifaceted Impact of SDS as a Minor Component

Mahbubur Rahman, Stephanie Ahmad, James Tarabokija, Nathaniel Parker and Ecevit Bilgili

S.1. Details of the DSC Method and Analysis of the DSC Traces of the Physical Mixtures

DSC of the as-received GF, Sol, HPC, physical mixtures (PMs), and spray-dried powders was performed using a Mettler-Toledo polymer analyzer (PolyDSC, Columbus, OH, USA) with integrated STARe 10 software. Around 6.0–7.0 mg of the powder sample was placed in an aluminum pan with a hole in the lid and loaded into the DSC machine. As-received GF was heated at a rate of 10 °C/min from 25 °C to 250 °C. All other samples were heated from 25 °C to 70 °C and the temperature was held for 2 min at 70 °C, then cooled back to 25 °C to remove any residual solvent in the sample. In the final step, the samples were heated from 25 °C to 250 °C at 10 °C/min. Nitrogen gas was used as the purge gas and protective gas at a flow rate of 50 mL/min and 150 mL/min, respectively. Figure S1 presents the DSC traces of the PMs and Table S1 presents the information about the thermal events observed during the DSC scans.

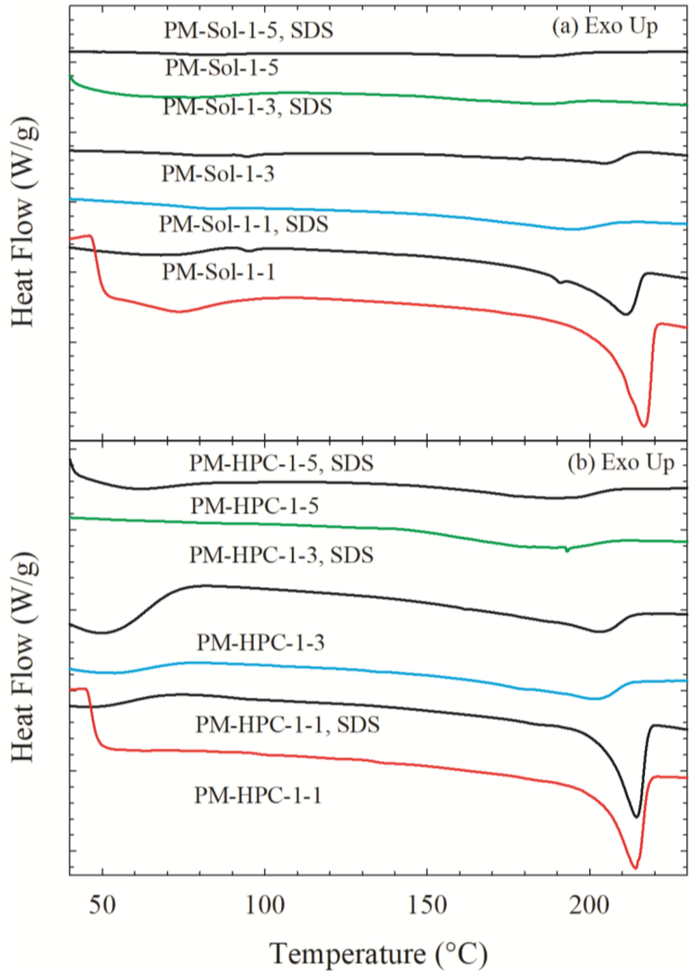

Figure S1. DSC traces of the physical mixtures (PMs) of GF–HPC/Sol with 1:1, 1:3, and 1:5 drug: polymer mass ratios and SDS and without SDS: (**a**) PMs with Sol and (**b**) PMs with HPC.

Table S1. Glass transition temperature, melting point temperature, and fusion enthalpy of the physical mixtures (PMs) obtained from DSC traces.

Formulation [a]	T_g (°C) [b]	T_m (°C) [b]	ΔH_f (J/g) [b]	ΔT_m (°C) [b]
PM-Sol-1:5	73.1	191	1.71	29.1
PM-Sol-1:3	78.3	198	7.23	22.1
PM-Sol-1:1	79.1	217	48.4	3.10
PM-Sol-1:5, SDS	76.7	190	1.56	30.1
PM-Sol-1:3, SDS	78.8	204	11.9	16.1
PM-Sol-1:1, SDS	77.3	212	29.1	8.10
PM-HPC-1:5	ND	193	11.3	27.1
PM-HPC-1:3	ND	205	14.6	15.1
PM-HPC-1:1	ND	215	40.2	5.10
PM-HPC-1:5, SDS	ND	194	7.61	26.1
PM-HPC-1:3, SDS	ND	205	14.1	15.1
PM-HPC-1:1, SDS	ND	214	35.0	6.10

[a] Sol denotes Soluplus; the ratios refer to the drug: polymer mass ratios. [b] T_m stands for the melting point temperature, T_g stands for the glass transition temperature, ΔT_m stands for the melting point depression, and ΔH_f stands for the fusion enthalpy.

S.2. Details of the Characterization Methods Used for Drug Wettability

Penetration of a liquid into a packed powder bed inside a cylindrical column allows for measurement of the powder wettability, based on the Washburn method [1,2]. The method presented here was adapted from Bilgili et al. [3] and Li et al. [4]. In the current study, powder and liquids, respectively, refer to GF (griseofulvin) powder and GF-saturated aqueous solutions of 15% Soluplus (Sol)/HPC with or w/o 0.125% SDS and 0.125% SDS alone. All percentages are (% *w*/*w*) with respective to deionized water. This polymer concentration was selected in order to measure the viscosity accurately in our viscometer set-up instead of the maximum viscosity of 12.5% used in the feed solutions. The solutions and deionized water were saturated with griseofulvin (GF) and stirred overnight. The saturated solutions were used for further characterization.

S.2.1. Apparent Shear Viscosity of the Solutions

The apparent shear viscosity of the solutions was measured using an R/S Plus Rheometer (Brookfield Engineering, Middleboro, MA, USA) with a water jacket assembly Lauda Eco (Lauda-Brinkmann LP, Delran, NJ, USA). A coaxial cylinder (CC40) was used to provide a controlled shear rate on the samples and shear rate from 0 to 1000 1/s for 60 s was used for all samples. The temperature of the jacket was kept constant at 25 ± 0.5 °C. The raw data were analyzed using the Rheo 3000 software (Brookfield Engineering, Middleboro, MA, USA) of the equipment to obtain the apparent shear viscosity as a function of the shear rate. The apparent shear viscosity at ~100 1/s was used as a representative low shear rate value. The viscosity of water and SDS solution were taken from Korson et al. [5] and Kushner et al. [6], respectively.

S.2.2. Surface Tension of the Solutions

The surface tension of the GF-saturated deionized water and the GF-saturated aqueous solutions of the polymer/surfactant was measured using an Attension Sigma 700 (Biolin Scientific, Linthicum, MD, USA). The Attension software calculates surface tension from force measurements of interaction of the probe (Wilhelmy plate) at the boundary between air and a liquid.

S.2.3. Drug Wettability with the Solutions

The Attension Sigma 700 set-up (Biolin Scientific, Linthicum, MD, USA) was used to study the penetration of GF-saturated deionized water/GF-saturated aqueous solutions of the polymer/surfactant into a packed powder bed of GF inside a cylindrical column and determine the

GF wettability, based on the modified Washburn method. The assembly consists of a sample holder in the form of a cylindrical metallic tube with small holes at the bottom as well as a hook at the top of the cover equipped with screw threads. About 0.8 g of GF powder was packed uniformly into the tube before each measurement. A filter paper was placed at the perforated end of the sample holder to support the GF powder sample. A petri dish containing deionized water or polymer/surfactant solution was placed below the perforated end of the holder on the mechanical platform.

Upon contact of the sample holder with the liquid, the liquid penetrated the GF powder bed, while the Attension Sigma 700 recorded the mass M of the liquid penetrated as a function of time T. The cosine of the contact angle θ for the GF-saturated deionized water/GF-saturated aqueous polymer/surfactant solution and drug can be determined using the modified Washburn equation, which provides a relationship between the mass of liquid penetration and contact angle, via

$$M^2 = \left(\frac{C\rho^2\gamma\cos\theta}{\eta}\right)T \tag{S1}$$

where η, ρ, and γ stand for viscosity of the liquid, density of the liquid, and surface tension of the liquid, respectively. C is a characteristic parameter of the powder sample, which could have been determined independently using a completely wetting liquid such as hexane, heptane, etc. Since the same drug powder (GF) was used as the powder sample and C depends only on the powder's packing-particle size, C remained invariant for different liquids studied here. This allows us to calculate the ratio of $\cos\theta_s/\cos\theta_w$ as a wetting effectiveness factor from the slopes of M^2 vs. T for deionized water and the polymer/surfactant solution. Here, θ_s is the contact angle between GF and the polymer–surfactant solution and θ_w is the contact angle between GF and deionized water. The wettability enhancement upon the use of different polymers/surfactant on the wetting of GF particles can be assessed by using this ratio, taking the wettability by water as a basis for comparison.

Experimental liquid penetration data (M^2 vs. T) for various liquids are presented in Figure 7 of the main text. The slope of the modified Washburn equation, i.e., $\frac{C\rho^2\gamma\cos\theta}{\eta}$, was obtained by fitting the linear region of the liquid penetration curve. The initial ~20 s was not considered due to transient behavior; data points that deviated from the linear region were also excluded. The modified Washburn equation fitted the data well ($R^2 \geq 0.98$). Using the slope for the different polymer/surfactant solutions and water, $\cos\theta_s/\cos\theta_w$ was calculated separately for each solution. The viscosity, surface tension, and calculated wetting effectiveness factor are reported in Table S2.

Table S2. Properties of deionized water and aqueous polymer–SDS solutions as well as the wetting effectiveness factor determined using the modified Washburn method.

Liquid [a]	η (cP)	ρ (g/mL)	γ (mN/m)	Slope, (g²/s) [b]	R^2 [b]	$\cos\theta_s/\cos\theta_w$ [b]
Water	0.89	1	66.5	7.0×10^{-3}	0.990	1
SDS	0.94	1.00	37.1	7.2×10^{-3}	0.975	1.94
Sol	8.76	1.01	41.4	1.2×10^{-3}	0.989	2.65
Sol–SDS	13.5	1.01	40.5	1.5×10^{-3}	0.991	4.65
HPC	53.2	1.01	39.9	1.5×10^{-3}	0.998	20.9
HPC–SDS	58.3	1.01	34.8	2.4×10^{-3}	0.999	42.1

[a] Saturated with GF. [b] Obtained from the line representing the liquid penetration data (refer to Figure 7 of the main text).

S.3. X-Ray Powder Diffractograms of SDS and As-Received Griseofulvin

Three intense non-overlapping peaks were detected at diffraction angles of 5.6°, 6.8°, and 8.3° in the SDS diffractogram (Figure S2, peaks shown in the orange dashed rectangle).

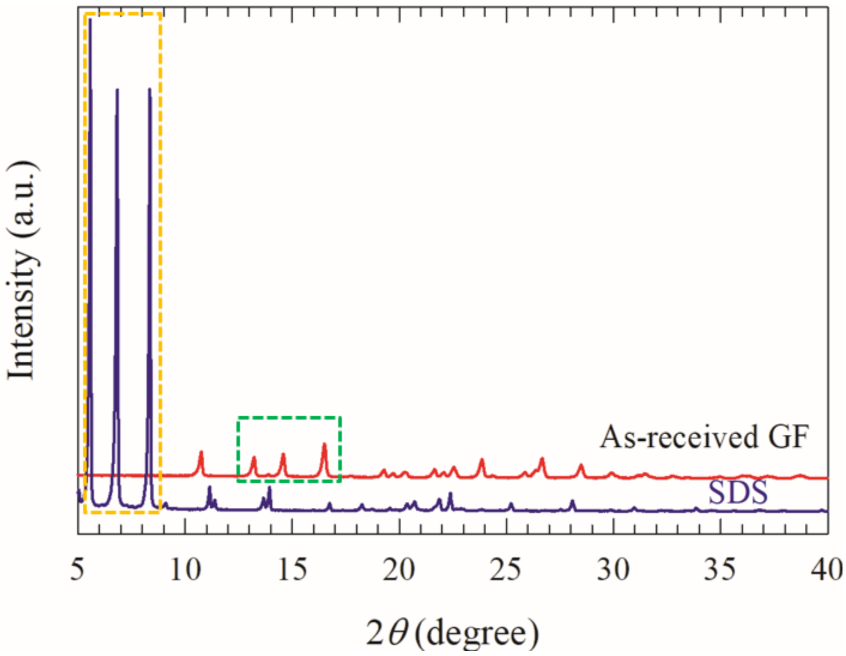

Figure S2. X-ray diffractograms of as-received GF and SDS powders.

We used three intense non-overlapping peaks at diffraction angles of 13.2°, 14.6°, and 16.5° in the GF diffractogram (the peaks shown in the green dashed rectangle), which do not interfere with the characteristic peaks of SDS, to calculate GF crystallinity. It should be noted that as SDS is a minor component of the spray-dried powders (0.8%–2.4% *w*/*w*), its peaks were not as intense as GF peaks in the diffractograms of the PMs (refer to Figure 2 of the main text).

References

1. Washburn, E. W., The dynamics of capillary flow. *Phys. Rev.* **1921,** *17* (3), 273–283.
2. Hołownia, D.; Kwiatkowska, I.; Hupka, J., An investigation on wetting of porous materials. *Physicochem. Prob. Miner. Process.* **2008,** *42*, 251–262.
3. Bilgili, E.; Rahman, M.; Palacios, D.; Arevalo, F., Impact of polymers on the aggregation of wet-milled itraconazole particles and their dissolution from spray-dried nanocomposites. *Adv. Powder Technol.* **2018,** *9* (12), 2941–2956.
4. Li, M.; Ioannidis, N.; Gogos, C.; Bilgili, E., A comparative assessment of nanocomposites vs. amorphous solid dispersions prepared via nanoextrusion for drug dissolution enhancement. *Eur. J. Pharm. Biopharm.* **2017,** *119*, 68–80.
5. Korson, L.; Drost-Hansen, W.; Millero, F. J., Viscosity of water at various temperatures. *J. Phys. Chem.* **1969,** *73* (1), 34–39.
6. Kushner, L. M.; Duncan, B. C.; Hoffman, J. I., A viscometric study of the micelles of sodium dodecyl sulfate in dilute solutions. *J. of Res. of the Nat. Bureau of Stand* **1952,** *49* (2), 85–90.
